# Garrett on Medical Electricity

**Published:** 1860-06

**Authors:** 


— A new book by Dr. Alfred C. Garratt, of Boston, is soon to be issued by Ticknor & Fields, of the same city. It is entitled the *Medical Uses of Electricity in the Treatment of Nervous Affections.* It will be a thoroughly systematic work, of over 700 pages, and nearly 100 cuts, showing not only the best methods for the therapeutical employment of electricity in the various nervous affections, but also showing the anatomy of the parts to be involved, and a concise view and means of diagnosis of the great variety of nervous diseases met with every day. The work proposes to fill a vacancy which exists in medical literature upon this subject.

